# 

**Published:** 1888-01

**Authors:** 


### CONTENTS.

ORIGINAL COMMUNICATIONS—

After Treatment of Cataract Extractions and Iridectomies. By R. O. Cotter, M. D., Macon, Ga. ..... 645

Diphtheria.—Its Cause and Treatment. By L. H. Jones, M. D., Atlanta, Ga. ..... 647

Treatment of Pneumonia. By C. B. Leitner, M. D., Flora, Ala. 652

Gonorrhœa Speedily Relieved. By J. J. Buster, M. D., Mt. Willing, S. C. ..... 654

The Relation of Intemperance to Insanity. By Clark Bell, Esq., New York ..... 655

CLINIC REPORTS—

Superinvolution of the Uterus Following Trachelorraphy. By Virgil O. Hardon, M. D., Atlanta, Ga. ..... 663

SOCIETY REPORTS—

The Clinical Society of Maryland ..... 667

Baltimore Academy of Medicine ..... 672

CORRESPONDENCE—

Our New York Letter ..... 680

EDITORIAL—

Chronic Alcoholism ..... 684

Items ..... 687

